# Pharmaceuticals Promoting Premature Termination Codon Readthrough: Progress in Development

**DOI:** 10.3390/biom13060988

**Published:** 2023-06-14

**Authors:** Shan Li, Juan Li, Wenjing Shi, Ziyan Nie, Shasha Zhang, Fengdie Ma, Jun Hu, Jianjun Chen, Peiqiang Li, Xiaodong Xie

**Affiliations:** 1 School of Basic Medical Sciences, Lanzhou University, Lanzhou 730000, China; lish2019@lzu.edu.cn (S.L.); shiwj21@lzu.edu.cn (W.S.); niezy20@lzu.edu.cn (Z.N.); shshzhang21@lzu.edu.cn (S.Z.); mafd20@lzu.edu.cn (F.M.); 220220926741@lzu.edu.cn (J.H.); 2 Central Laboratory, The First Hospital of Lanzhou University, Lanzhou 730000, China; lj67629@163.com; 3 Gansu Key Laboratory of Genetic Study of Hematopathy, The First Hospital of Lanzhou University, Lanzhou 730000, China; 4 State Key Laboratory of Applied Organic Chemistry, College of Chemistry and Chemical Engineering, Lanzhou University, Lanzhou 730000, China; chenjj@lzu.edu.cn

**Keywords:** nonsense mutation, premature termination codon (PTC), translational readthrough-inducing drugs (TRIDs), nonsense suppression, readthrough therapy

## Abstract

Around 11% of all known gene lesions causing human genetic diseases are nonsense mutations that introduce a premature stop codon (PTC) into the protein-coding gene sequence. Drug-induced PTC readthrough is a promising therapeutic strategy for treating hereditary diseases caused by nonsense mutations. To date, it has been found that more than 50 small-molecular compounds can promote PTC readthrough, known as translational readthrough-inducing drugs (TRIDs), and can be divided into two major categories: aminoglycosides and non-aminoglycosides. This review summarizes the pharmacodynamics and clinical application potential of the main TRIDs discovered so far, especially some newly discovered TRIDs in the past decade. The discovery of these TRIDs brings hope for treating nonsense mutations in various genetic diseases. Further research is still needed to deeply understand the mechanism of eukaryotic cell termination and drug-induced PTC readthrough so that patients can achieve the greatest benefit from the various TRID treatments.

## 1. Introduction

Around 11% of all described gene lesions are nonsense mutations resulting in a UGA, UAG, or UAA premature stop codon (PTC) [1]. In some cases, PTC-containing mRNAs are committed to rapid decay through the nonsense-mediated mRNA decay (NMD) pathway. NMD is a translation-dependent mRNA quality surveillance mechanism that prevents the accumulation of potentially deleterious truncated proteins produced by PTC, regulated by Upf1, Upf2, and Upf3 proteins. Some mRNAs carrying a PTC can escape NMD and translate into truncated, often dysfunctional, proteins that can have a dominant-negative or gain-of-function effect on gene function. NMD efficiency varies across transcripts depending on the position of the PTC, tissue type, oncogenesis, and stress conditions [2].

Eukaryotes require eRF1 to enter the A site of the ribosome to recognize the stop codon to terminate protein translation, but this process is not 100% efficient. Under certain conditions, a near-cognate tRNA (nc-tRNAs) can be recruited to the A site of the ribosome to outcompete eRF1 by base-pairing two out of the three anticodon bases to the stop codon in the mRNA. This condition results in the nc-tRNAs accommodating in the A site of the ribosome, misinterpreting the stop codon as a sense codon and causing translation to continue until it reaches the later stop codon, the so-called readthrough of the stop codon. Several reviews have recently discussed molecular factors regulating translation termination and stopping codon readthrough in higher eukaryotes [3,4].

Without any readthrough molecules, the readthrough of any physiological stop codon or PTC is referred to as basal readthrough. At the natural stop codon, the basal readthrough efficiency in mammalian species ranges between 0.01 and 0.1%, except for programmed readthrough targeting specific mRNAs [5]. PTC basal readthrough levels are higher and can vary from 0.01% to even 1%, probably because PTC is generally farther from the 3′UTR than the normal stop codon, limiting the interaction between eRFs and PABPs, resulting in less efficient eRF3 action and delayed release of the transcript from the ribosome [6,7]. PTC basal readthrough generally occurs on the fraction of PTC-carrying mRNAs that escape NMD. Although the amount of protein generated by basal readthrough is minute, it has the potential to partially restore protein functionality. For example, the clinical phenotype of patients who escape NMD and develop basal readthrough appears to be milder than that of patients who fully experience NMD, even if they carry the same nonsense mutation [3,8]. Another type of stop codon readthrough is PTC readthrough promoted by specific molecules, termed induced readthrough, which is a promising approach to treating genetic diseases and cancers caused by nonsense mutations [4]. When the ribosome reaches a PTC, the presence of such molecules favors the recruitment of nc-tRNAs instead of the translation termination complex, thereby at least partially restoring full-length protein expression and function. A small amount of functional protein recovery may improve or slow the progression of disease in many recessive, loss-of-function disorders. Interestingly, the efficiency of induced readthrough may be related to the basal level of readthrough that occurs at PTC: the higher the level of basal readthrough, the more efficient the readthrough promoted by molecules [9,10].

**Figure 1:**
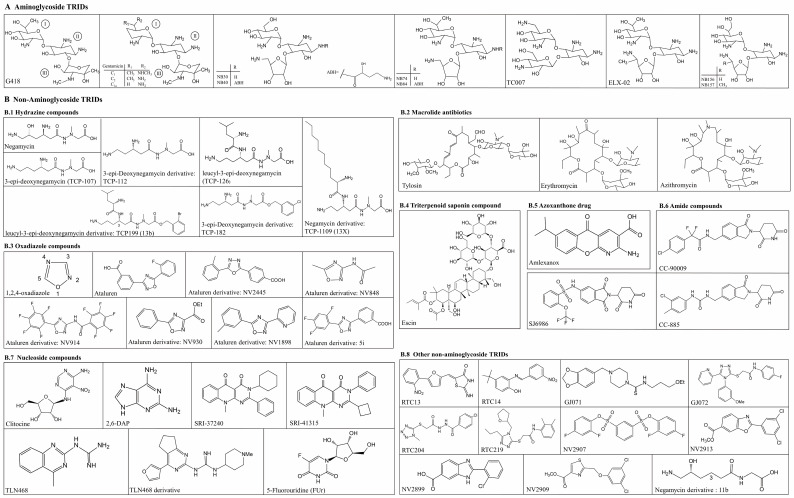
Chemical structures of the main TRIDs: including (**A**) aminoglycosides and (**B**) non-aminoglycosides.

PTC readthrough therapy, also known as nonsense suppression therapy, offers many advantages over gene replacement/editing therapy in treating genetic disorders. First, there is no alteration to the endogenous control of the targeted gene, tissue specificity, timing, and duration of expression. Second, the problems of being off-target, pathogenic gene size, and the human body’s immunity to the vector do not exist. Finally, readthrough therapy is not gene-specific and thus allows for treating diverse genetic conditions [11]. Currently, several methods have been developed to induce PTC readthrough, including molecules activating PTC readthrough, suppressor tRNAs, pseudouridylation, and RNA editing [4,12], with small molecules being the focus of many studies due to their potential therapeutic interest. In 1985, it was first discovered that the aminoglycosides G418 and paromomycin could promote the readthrough of PTCs [13]. More aminoglycosides and non-aminoglycosidic molecules have been shown to possess similar properties since then. To date, it has been found that more than 50 small-molecular compounds can promote PTC readthrough, known as translational readthrough inducing drugs (TRIDs). Some of these compounds have entered clinical trials or been marketed due to their excellent PTC readthrough effect in cell-based assays, cultured mammalian cells, animal models, and even human patients with nonsense mutations, while some compounds are toxic, and adverse side effects limit their potential clinical uses. This review summarizes the structure (Figure 1), pharmacodynamics, and clinical application potential of the main TRIDs discovered so far, especially some newly discovered TRIDs in the past decade.

## 2. Aminoglycosides TRIDs

Aminoglycosides are a class of structurally related antibiotics of low molecular weight (300–600 Da) consisting of a 2-deoxystreptamine ring linked to multiple amino sugars [7]. These compounds tightly bind to the decoding center of the bacterial ribosome at low doses, resulting in effective misincorporation of the nc-tRNAs and extensive codon misreading [14]. These antibiotics act by inhibiting translation initiation and by interfering with bacterial protein synthesis at high doses [4]. At high doses, they lead to the inhibition of translation initiation and interfere with bacterial protein synthesis [4]. In eukaryotic ribosomes, these antibiotics are less effective at binding than bacterial ribosomes due to differences at two critical nucleotides of the rRNA nucleotide sequence [15]. Nonetheless, in the presence of a PTC, the impact of aminoglycosides on the eukaryotic translational apparatus is generally sufficient to cause the misincorporation of nc-tRNAs at the A site to induce PTC readthrough [6,7]. In 1985, Burke and Mogg first discovered that aminoglycosides G418 and paromomycin could induce PTC readthrough in COS-7 cells [13]. Since this breakthrough, other members of the aminoglycosides family, e.g., gentamicin, neomycin, kanamycin, amikacin, arbekacin, and lividomycin have also been demonstrated to show different PTC readthrough efficacies in vitro and/or in vivo systems. Still, not all aminoglycosides exhibit this effect. For example, hygromycin and streptomycin did not exhibit a substantial increase in readthrough levels. This discrepancy could potentially be attributed to the variances in their chemical structures and their varying affinity for the decoding centers of mammalian ribosomes [10]. PTCs are suppressed more effectively by aminoglycosides containing a C6′ hydroxyl group ring I, such as G418 or paromomycin [16]. Furthermore, the PTC readthrough efficiency of an aminoglycoside is also dependent on PTC identity, PTC sequence contexts, and other factors [17]. A recent study showed that TRPC nonselective cation channels might contribute to aminoglycosides’ variable PTC readthrough effect by controlling cellular uptake [18]. Structural analysis of the complex of yeast 80S ribosomes with paromomycin, G418, gentamicin, and TC007 revealed that aminoglycosides interacted with the eukaryotic ribosome at multiple sites and aminoglycosides with different structures may promote PTC readthrough by distinct mechanisms [19]. Here, we mainly introduce the two most common aminoglycosides, G418, and gentamicin, in preclinical studies of PTC readthrough.

### 2.1. G418

G418, also known as geneticin, is produced by *Micromonospora rhodorangea* and is commonly used in laboratory research to select genetically modified cells (not used clinically as an antibiotic) [20]. It belongs to the 4,6-linked aminoglycoside class that contains a ring I 6′-OH group. This structure is likely to bind to the pocket within helix 44 of the eukaryotic ribosome, facilitating nc-tRNAs to be effectively accommodated in the A site to compete with the termination factors, thereby inducing PTC readthrough [19]. The readthrough efficiency of G418 increased in a concentration-dependent manner, but high doses of G418 were cytotoxic [17]. In addition, G418 can increase mRNA levels containing PTC, possibly because the readthrough of PTC displaces RNA binding proteins (such as the exon-junction complex) to protect the mRNA from NMD [21]. The ability of G418 to induce the PTC readthrough was investigated in a variety of nonsense mutation disease models summarized in Supplementary Table S1, which can induce variable levels of PTC readthrough in many disease models. Notably, Wangen et al. found through ribosome profiling that aminoglycoside, especially G418, not only stimulated the readthrough of PTCs, but also stimulated the readthrough of normal stop codons genome-wide in human cell lines, resulting in broad perturbation of several cellular processes, among which the translation of histone genes, selenoprotein genes, and S-adenosylmethionine decarboxylase (AMD1) was significantly affected by G418 treatment [22]. Therefore, although G418 is the strongest PTC readthrough inducer among all aminoglycosides tested to date, it is not suitable for clinical use as a readthrough agent.

### 2.2. Gentamicin

Gentamicin, first discovered in 1963, is used clinically to treat severe gram-negative antibacterial infections despite its potential safety concerns [20]. Produced by fermentation from *Micromonospora purpurea*, gentamicin is supplied as a mixture comprising the major components (gentamicins C1, C1a, C2, and C2a, accounting for 92–99% of the complex) and minor components (gentamicins A, B, B1, C2b, and X2, garosamine, sisomicin, 2-deoxystreptamine and garamine) [23]. Friesen. et al. found that each of the four major gentamicin complex components had similar PTC readthrough activity to the complex itself, and gentamicin X2 (a minor component) was the most potent and active readthrough component in the gentamicin complex with a better safety/readthrough potency ratio than gentamicin, G418 or NB124, in which gentamicin X2 is approximately 2-fold more potent than G418 [23,24]. Gentamicin belongs to the 4,6-linked aminoglycoside class that contains a 6′-NH2 constituent in ring I. This structure does not bind to helix h44, which may hamper the interaction between the release factor and ribosome through an alternative mechanism based on inter-subunit rotation effects, thus promoting PTC readthrough [19]. As with G418, gentamicin showed a dose-dependent readthrough effect, but it required a higher concentration than G418 to induce significant readthrough [9]. Gentamicin is less toxic than G418, and several clinical trials have shown that short-term intravenous gentamicin at 7.5–10 mg/kg/d can induce certain PTC readthrough without causing ototoxicity and nephrotoxicity [25,26]. The therapeutic potential of gentamicin as TRIDs has been tested in several pre-clinical readthrough models summarized in Supplementary Table S1 and many studies showed positive results. Furthermore, it has also been clinically studied in several PTC-carrying genetic disorders, including cystic fibrosis (CF), Duchenne muscular dystrophy (DMD), hemophilia, and genodermatosis. Gentamicin is the only aminoglycoside tested in clinical trials of PTC readthrough. Next, we mainly review the related clinical studies.

In CF, preliminary studies on cell and animal models have shown that gentamicin can induce PTC readthrough of the CFTR gene, resulting in the synthesis of a full-length and functional CFTR protein [27], followed by several clinical studies investigated the potential benefit of gentamicin treatment in CF patients with nonsense mutations. Only a small percentage of CF patients treated with the topical nasal application of gentamicin or systemically by intravenous administration had increased CFTR-dependent chloride transport in epithelial cells [28], which improved respiratory function and decreased sweat chloride level [29]. Some CF patients do not respond to gentamicin therapy, which might be related to their own genetic background. However, the gentamicin-induced change did not persist after discontinuation of treatment [29], so long-term administration seems necessary. The safety and efficacy of long-term gentamicin use need to be further evaluated.

In 1999, researchers demonstrated in an *mdx* mouse model of DMD that gentamicin can promote PTC readthrough of the *dmd* gene to generate a full-length dystrophin protein [30], which led to several clinical trials using an intravenous application of gentamicin in DMD patients [31,32,33]. An increase in dystrophin protein in muscle biopsies and a decrease in serum creatine kinase were observed in a portion of DMD patients treated with gentamicin, but clear clinical efficacy was not achieved [32,33].

Furthermore, a clinical trial evaluated the potential therapeutic benefit of gentamicin for hemophilia patients. After intravenous injection of gentamicin for three consecutive days, two patients out of five patients enrolled showed changes in hemostatic indexes, but the changes were very small. Given its potential toxicity, the authors considered that gentamicin was unlikely to be an effective drug for the treatment of severe hemophilia carrying nonsense mutations [34].

In general, the transdermal delivery of gentamicin is considered unfeasible because of its low permeability through the dermis [35]. However, topical gentamicin is a very promising treatment option for genodermatosis caused by nonsense mutations, which is not only low in toxicity but also convenient and inexpensive. The daily topical dose of gentamicin is much lower than the systemic dose used in clinical studies [36]. Clinical studies of topical gentamicin treatment have been performed in four different genodermatosis, including Nagashima-type palmoplantar keratosis (NPPK) [37,38], different subtypes of epidermolysis bullosa [39,40,41,42], Hailey-Hailey disease (HHD) [43] and hereditary hypotrichosis simplex of the scalp (HSS) [44], all enrolled patients benefited from treatment without significant side effects (Table 1). Although chronic exposure to topical gentamicin could enhance bacterial resistance, it is not comparable with the benefits [36]. Interestingly, the treatment of genodermatosis by systemic gentamicin administration has also recently been reported, mainly involving different subtypes of epidermolysis bullosa [25,26,45]. After short-term intravenous gentamicin, the patient’s quality of life has improved. The long-term safety and efficacy need to be further evaluated.

The stimulation of PTC readthrough is a transient phenomenon that usually requires lifetime use of the drugs. Studies in animal models showed that aminoglycosides work in a peak-driven way, and gentamicin administered continuously at low concentrations did not cause readthrough [30]. Thus, the primary issue impeding the long-term clinical use of aminoglycosides as readthrough agents are the narrow therapeutic window between inducing sufficient PTC readthrough and causing ototoxicity, nephrotoxicity, and/or retinal toxicity [4,10]. Several approaches to reduce aminoglycoside toxicity are to date being explored, such as co-administration with antioxidants, poly-l-aspartate, daptomycin, or liposome encapsulation [6]. Recently, a new approach to reduce aminoglycoside toxicity has been proposed. Several studies have identified by high-throughput screening (HTS) that some molecules themselves do not have the effect of promoting PTC readthrough, but can potentiate the readthrough activity of aminoglycosides, which makes it possible to significantly lowering of the therapeutic dose of aminoglycosides, thus reducing the aminoglycosides-related toxicity. Currently discovered potentiators of aminoglycosides include phthalimide derivatives (CDX series, e.g., CDX5-1 increases about 180-fold the readthrough efficiency of G418 in human cells) [46], 2-aminothiazole-4-carboxamide derivatives [47], antimalarial drug mefloquine [48] and Y-320 [49], but how these molecules improve the readthrough efficiency of aminoglycosides is unclear.

### 2.3. Aminoglycoside Derivatives

Aminoglycoside-mediated toxicity is due primarily to their combination with off-target sites, such as lysosomal membranes and mitochondrial ribosomes [4], and does not appear to be related to their ability to induce PTC readthrough. Based on the hypothesis that the structural elements of aminoglycosides that cause toxicity can be separated from those inducing PTC readthrough [50], researchers have developed a batch of aminoglycoside derivatives with reduced toxicity and retained or increased readthrough activity through reasonable modification/design of aminoglycoside structure.

#### 2.3.1. NB30 and NB54

The aminoglycoside derivatives of the first generation (NB30) and the second generation (NB54) were developed by optimizing the structure-activity-toxicity relationship of the paromamine scaffold [51]. Both exhibited lower toxicity and antibacterial activity than gentamicin and paromomycin. They have been demonstrated to be able to induce the readthrough of PTC in disease models of Usher syndrome (USH) [11,52], CF [53], Rett syndrome [54], and mucopolysaccharidosis type I-Hurler (MPS I-H) [55], and NB54 induced readthrough more effectively than those of gentamicin, paromomycin and NB30 [11,51].

#### 2.3.2. NB74 and NB84

Modifying the G418 structure resulted in the third generation of aminoglycoside derivatives, NB74 and NB84, which had cytotoxicity similar to NB30 and NB54 [56]. NB84 exhibited superior readthrough activity than gentamicin and previous generations of aminoglycoside derivatives, as demonstrated in several models of Rett syndrome and MPS I-H [54,55]. Long-term (28-weeks) treatment of MPS I-H mice with NB84 showed a recovery of α-L-iduronidase activity and a reduction in glycosaminoglycan accumulation, ameliorating MPS I-H progression in multiple tissues, including the brain, heart, and bone, with no visible toxicity was observed [57]. Notably, results for brain tissue indicated that NB84 could pass the blood-brain barrier, suggesting good availability of designer aminoglycosides [57].

#### 2.3.3. TC007

TC007 was a derivative of neomycin and belonged to the class of pyranmycins, which have comparable antibacterial activity to neomycin but exhibit much-improved acid stability [58]. It has been identified as a potential SMNΔ7 stop codon readthrough agent for spinal muscular atrophy (SMA). TC007 restored the levels of SMN protein in fibroblasts and astrocytes differentiated from induced pluripotent stem cells (iPSCs) of SMA patients [59]. Furthermore, in SMAΔ7 mice, direct delivery of TC007 to the central nervous system significantly elevated the expression of SMN protein in the brain and spinal cord and increased the lifespan (approximately 30%) of the mice [60]. In contrast, the delivery of TC007 by subcutaneous injection did not result in a significant increase in SMN protein, nor did it enhance the lifespan of SMA mice, however, showed an improvement in gross motor function [61]. Thus, in the case of non-syndromic disorders, it is crucial to prioritize targeted delivery of TRIDs to the affected organ or specific tissue as a fundamental aspect of PTC readthrough therapy.

#### 2.3.4. ELX-02

ELX-02, previously referred to as NB-124, is a novel synthetic aminoglycoside derivative that exhibits high selectivity toward the eukaryotic ribosome and decreases binding to mitochondrial and prokaryotic ribosomes compared to aminoglycosides, which results in reduced antibacterial activity (a 100-fold lower than gentamicin) and toxicity, and significantly increased readthrough activity [62]. It is considered a promising non-toxic alternative to G418. ELX-02 induced *TP53* and *APC* PTC readthrough with significantly higher efficiency than gentamicin (2–5 times higher) [63]. In multiple CF preclinical models, ELX-02 significantly rescued CFTR function with a ten times improvement in the therapeutic index over gentamicin and other aminoglycoside derivatives, and CFTR activity could be further augmented by the addition of the CFTR potentiator, corrector, or NMD inhibitor [64,65]. Furthermore, ELX-02 restored functional CTNS protein expression in cystinosis cell and animal models, as evidenced by reduced cystine accumulation [66]. Several studies have shown that ELX-02 can stabilize PTC-containing mRNA transcripts and promote PTC readthrough in a dose-dependent manner, similar to G418 [62,64,66]. However, ELX-02 does not promote the readthrough of normal stop codons at concentrations that lead to PTC readthrough [67]. To date, three Phase I clinical trials (NCT03292302, NCT03776539, NCT03309605) have been completed to evaluate the safety, tolerability, and pharmacokinetics of ELX-02 in healthy adult volunteers and renal impaired population, with results indicating that ELX-02 was generally well tolerated in the subject population, no reported drug-related serious adverse events or nephrotoxicity and limited, reversible, auditory findings [68,69,70]. ELX-02 is poorly absorbed orally and nearly 100% bioavailable when administered subcutaneously [68]. It is excreted into the urine primarily as a parent compound via the kidneys, and its pharmacokinetic properties are similar to those of aminoglycoside antibiotics such as gentamicin [68,70]. Interestingly, the defined relationship between eGFR (estimated glomerular filtration rate) and plasma exposure based on the Phase I renal impairment trial (NCT03776539) made possible targeted individualized dosing of ELX-02 in patients with nephropathic cystinosis [69]. Four Phase II clinical trials with ELX-02 have recently commenced, including two in CF (NCT04126473, NCT04135495), one in nephropathic cystinosis (NCT04069260), and, one in Alport syndrome (NCT05448755). NCT04069260 was terminated early due to study design limitations. NCT04135495 was completed and study results have not yet been published.

#### 2.3.5. Other Aminoglycoside Derivatives

Recently, the aminoglycoside derivatives NB156 and NB157 have been designed and synthesized by researchers. They differ from NB74 and ELX-02 in that there are additional hydroxyl groups on the side-chain of the glucosamine ring (ring I) and show significantly higher readthrough activity than the parent structures [71]. Lee et al. found that the 3′,4′-dihydroxy, and 6′-methyl scaffolds were indispensable for the PTC readthrough activity of aminoglycosides. They synthesized five aminoglycoside analogs, among which a kanamycin analog (6′-methyl-3″-deamino-3″-hydroxykanamycin C) showed higher readthrough activity than gentamicin in the CF cell model [72].

## 3. Non-Aminoglycoside TRIDs

### 3.1. Hydrazine Compounds—Negamycin and Its Derivatives

Negamycin is a natural dipeptide antibiotic with a hydrazide structure, exhibiting efficacious antimicrobial activity against Gram-negative microorganisms. It is less toxic than gentamicin but has never been approved for clinical use. The ability of negamycin to induce PTC readthrough has been confirmed in models of DMD, congenital muscular dystrophy (CMD), CF, and APC tumor suppressor gene [73]. Additionally, negamycin administration also effectively inhibited PTC-containing mRNA degradation in CMD patient-derived cell cultures. One study found that, similar to aminoglycosides, negamycin could bind to a partial sequence of the eukaryotic rRNA-decoding A-site [74], but it has also been reported to bind the polypeptide exit tunnel in the 50S ribosomal subunit [75]. The specific mechanism of negamycin-induced PTC readthrough is not known. Interestingly, two natural products related to negamycin (3-epi-deoxynegamycin and leucyl-3-epideoxynegamycin) that did not display antimicrobial activity were also found to be potent PTC readthrough agents [76]. The structure-activity relationship (SAR) of the above three compounds was further studied, and several derivatives, including 11b, TCP112, TCP182, TCP199, and TCP1109 [77] showed better readthrough activity than the parent compound. A recent study found that TCP-1109 increased the production of the myelin protein zero readthrough isoform (L-MPZ) in vitro and in vivo [78], indicating that it altered the programmed translational readthrough, which may be detrimental to PTC readthrough therapy.

### 3.2. Macrolide Antibiotics—Tylosin, Erythromycin and Azithromycin

**Figure 2:**
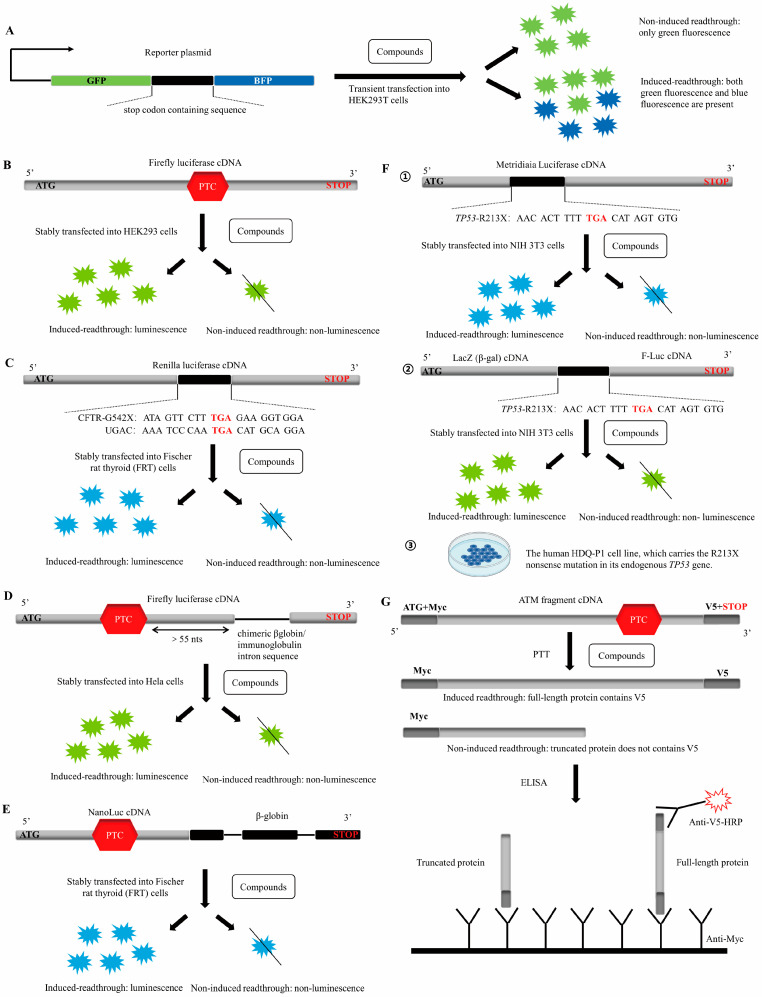
Currently used screening systems to identify TRIDs. (**A**) A PTC-containing sequence is introduced between the green fluorescent protein (GFP) and the blue fluorescent protein (BFP) open reading frames. Only GFP-labeled cells are collected for analysis and an increase in the BFP-mean fluorescence intensity indicated that the compound has the ability to readthrough [81]. (**B**,**C**) The PTC or PTC-containing sequence is introduced into the cDNA encoding the luciferase, and the luminescence is detected indicating that the compound induces readthrough, possibly with off-target effects [85,93]. (**D**,**E**) The intron is introduced into the cDNA sequence of luciferase carrying PTC, which subjects the corresponding mRNA to splicing and/or NMD. Luminescence is detected indicating that the compound simultaneously inhibits NMD and activates the readthrough or very strongly activates the readthrough of PTC-mRNAs escaping NMD [94,95]. (**F**) Three-step workflow strategy based on complementary assays. ① Similar to (**B**,**C**); ② Use of two cDNAs encoding β-galactosidase or luciferase separated by a PTC-containing sequence. The β-galactosidase is used as an internal control for the normalization, and the luminescence is detected indicating that the compound induces readthrough; ③ Human HDQ-P1 cell line, which carries the R213X nonsense mutation in its *TP53* gene. The full-length P53 is detected indicating that the compound induces endogenous PTC readthrough [96]. (**G**) PTT–ELISA. A PTC mutation of the ATM gene fragment is cloned into plasmids and tagged with c-myc and V5 epitopes at each end. Compounds with PTC readthrough activity induce the production of V5-containing full-length proteins, which can be identified by anti-V5-HRP [97].

Several macrolide antibiotics have also been proven to be effective readthrough agents in different PTC-mediated diseases, which may act by weakening the binding of the release factor or disrupting peptide release mechanisms [79]. Tylosin, with low oral toxicity and few side effects, induced the readthrough of a PTC in the APC gene and reduced xenograft tumor growth in nude mice [80]. The effect was also confirmed in Min mice (*Apc*^Min/+^) model-treatment with tylosin resulting in decreased intestinal polyp size and prolonged life span of the Min mouse [80]. In 2016, Caspi et al. developed a reporter plasmid based on two fluorescent proteins that allow rapid and efficient screening readthrough drugs (Figure 2A). Using this vector, they found that the macrolide antibiotic erythromycin and its derivative azithromycin can induce the readthrough of nonsense mutations. Subsequently, the ability of erythromycin and azithromycin to induce PTC readthrough was demonstrated in patient-derived cells of ataxia-telangiectasia (A-T), RTT syndrome, and SMA [81]. Erythromycin has also been revealed to induce PTC readthrough of the *APC* gene and reduce intestinal polyp number in Min mice [81]. Notably, the concentration required for azithromycin to induce readthrough was 100-fold lower than erythromycin or gentamycin, which is beneficial to clinical treatment as lower doses of antibiotics are expected to lead to fewer side effects [81]. In the SMA mouse model, intracerebroventricular administration of azithromycin increased SMN protein in disease-related tissues but did not improve clinical phenotype. When azithromycin and SMN2 antisense oligonucleotides (ASO) were co-administered, the phenotypic improvement of SMA mice exceeded that of azithromycin or ASO alone [82]. A recent study found that tylosin and azithromycin can also stimulate the PTC readthrough of certain genes of primary ciliary dyskinesia, but the readthrough efficiency is lower than that of gentamicin and G418 [83].

### 3.3. Oxadiazole Compounds—Ataluren and Its Derivatives

Oxadiazole is a five-membered heterocyclic ring containing one oxygen and two nitrogen atoms, including 1,2,3-oxadiazole, 1,2,4-oxadiazole, 1,3,4-oxadiazole and 1,2,5-oxadiazole, which is the key component of pharmacophores and ligand binding [84]. Ataluren, also known as PTC124 or translarna™, is an oxadiazole compound (3-[5-(2-fluorophenyl)-1,2,4-oxadiazol-3-yl]-benzoic acid) discovered by PTC Therapeutics that promote PTC readthrough in mammalian cells.

Ataluren was identified as a readthrough agent in 2007 from ~800,000 low molecular weight compounds using two HTS systems, including HEK293 cells stably transfected with a Firefly luciferase (FLuc) reporter gene containing UGA in codon 190 (Figure 2B) and a cell-free translation system containing synthetic nonsense-containing LUC-190 mRNA and rabbit reticulocyte lysate) [85]. Subsequently, the authors further characterized the pharmacology of ataluren, which promotes readthrough of each of the three nonsense codons in stable cell lines harboring LUC-190 nonsense alleles, with the highest efficiency at UGA, followed by UAG and then UAA. Continuous exposure to effective ataluren concentrations is essential for maximizing and maintaining nonsense suppression because the readthrough of PTC by ataluren in cell-based assays was rapidly reversed upon drug withdrawal [85]. The initial study showed that ataluren had no significant effect on the levels of normal mRNAs or mRNAs that are endogenous NMD substrates, indicating no effects on either transcription or mRNA stability [85]. But several recent studies showed that ataluren could stabilize the level of PTC-containing mRNA [86,87]. Importantly, unlike aminoglycosides, nonsense suppression of ataluren appears selective to PTCs without affecting the normal stop codons [85]. A series of ataluren derivatives have been developed in which NV848, NV914, NV930 [88], NV2445 [89], NV1898 [90], PTC414 [91], and 5i [92] have similar readthrough activity to ataluren.

A recent series of studies have described the mechanism of action (MOA) of ataluren in inducing PTC readthrough. In 2016, Roy et al. found that ataluren’s likely target is the ribosome. It produces full-length protein by promoting the insertion of near-cognate tRNAs at the site of the nonsense codon without apparent effects on transcription, mRNA processing, mRNA stability, or protein stability [98]. In 2018, Ng et al. employed a highly purified, eukaryotic cell-free protein synthesis system to demonstrate that both aminoglycosides and ataluren stimulate readthrough by directly affecting the protein synthesis machinery, but with distinct mechanisms. Aminoglycosides induce readthrough by binding to a single tight site on ribosomes, while ataluren-like compounds induce a slower change in the protein synthesis apparatus by weaker, multisite binding, thus permitting readthrough [24]. In the subsequent experiments, they further demonstrated that ataluren and G418 stimulate readthrough by orthogonal mechanisms, suggesting the additivity of the combined action of these two TRIDs. Ataluren stimulation of readthrough was entirely attributed to its ability to suppress release factor complex (RFC, eRF1.eRF3.GTP) activity. In contrast, G418 binding to its high-affinity site on the ribosome increases functional near-cognate tRNA mispairing, with little effect on RFC activity [99]. Recently, they identified two sites of ataluren binding within rRNA by photoaffinity labeling, one near the ribosome decoding center and the other within the peptidyl transfer center. These two sites are directly responsible for ataluren inhibition of termination activity. A third site, within the RFC, has as yet unclear functional consequences [100]. Interestingly, Tutone et al. proposed another MOA of ataluren through an in silico compared study, that is, mRNA was the most likely candidate target of this compound and the binding of ataluren to mRNA can lead to mRNA:tRNA mispairing and provoke the insertion of a near-cognate tRNA [101].

Phase I clinical studies showed that ataluren had no apparent toxicity or adverse effects. It was palatable and well tolerated when administered orally as a liquid suspension through a single dose of 100 mg/kg. A series of subsequent clinical trials confirmed a favorable safety profile for ataluren. The primary in vivo metabolic pathways for ataluren in humans were direct conjugation with glucuronic acid, glycine, carnitine, oxidation, and oxadiazole ring cleavage products, and ataluren acyl glucuronide was the only detectable metabolite in human plasma. Ataluren efficiency depends on the dose used, and it showed a bell-shaped concentration–response curve [102].

To date, more than 50 publications have documented the PTC readthrough activity of ataluren in nearly 40 non-clinical models of genetic disorders caused by nonsense mutation. Initially, the readthrough activity of ataluren was confirmed in the *mdx* mice model and primary muscle cells from humans, which promoted dystrophin production and rescued striated muscle function in *mdx* mice. Patient-derived fibroblasts, induced pluripotent stem cells (iPSCs), and organoids are ideal models for studying the treatment of human diseases. In the last 10 years, the readthrough activity of ataluren was studied in the above models, which involved several hereditary diseases with nonsense mutations. It is found that ataluren has a good therapeutic effect on retinitis pigmentosa, Bardet-Biedl syndrome, Alström syndrome, Usher syndrome, and neuronal ceroid lipofuscinosis, but its therapeutic effect on Leber congenital amaurosis type 4, inherited retinal dystrophies, Lamin A/C (LMNA)-related cardiomyopathy, cystic fibrosis, choroideremia, and SCN5A nonsense mutation was limited [87,91,103,104,105,106,107,108,109,110,111,112,113] (Table 2), which may be related to PTC identity, PTC context sequence, and NMD efficiency. The readthrough activity of ataluren in other models can be found in Supplementary Table S1.

Due to the good readthrough activity in preclinical studies and the low toxicity, ataluren was further tested in human clinical trials, including CF, DMD, Dravet Syndrome (DS), CDKL5 Deficiency Syndrome (CDD), and aniridia. Next, we mainly introduce the relevant clinical research results.

PTC Therapeutics has decided to stop the clinical development of ataluren in CF due to the failure of Phase III clinical trials (NCT02139306) to achieve primary or secondary endpoints and withdrew CF’s application for marketing authorization in Europe [114].

48-week Phase IIb and III clinical trials of ataluren in DMD have been completed (Table 3). Although neither of them reached its primary endpoint, the post-hoc analysis showed that ataluren provides a slowing of disease progression compared with a placebo in patients with nonsense mutation DMD (nmDMD), especially in subgroups with a baseline 6-min walk distance of 300 m or more to less than 400 (the ambulatory transition phase) [115]. Patients aged 5 and older with ambulatory nmDMD were conditionally approved for the drug in the European Union (EU) in 2014. This approval is conditional on PTC Therapeutics completing the confirmatory Phase III trial in nmDMD and submitting additional efficacy and safety data from this trial [116]. As of 2018, the indication was extended to children who are able to walk between 2 and 5 years old [102]. Ataluren 40 mg/kg/day (10 mg/kg in the morning, 10 mg/kg at midday, and 20 mg/kg in the evening) is also conditionally approved for the treatment of nmDMD in ambulatory patients aged 2 years or older in Iceland, Norway, Liechtenstein, Belarus, Great Britain, Northern Ireland, Israel, Kazakhstan, Brazil, Russia, the Republic of Korea, Italy, Finland, The Netherlands, and Lithuania or aged 5 years or older in Chile and Ukraine (under special state registration in Ukraine) [117], whereas in the US, ataluren is an investigational drug. So far, the FDA has rejected ataluren’s application for DMD as promising results were obtained via post-hoc statistical and exploratory analyses, necessitating confirmatory clinical trials [102]. The long-term safety and efficacy of ataluren are being assessed in another Phase III clinical trial (NCT03179631), which is estimated to finish in July 2023. Strategic Targeting of Registries and International Database of Excellence (STRIDE; NCT02369731, started in April 2015) is an ongoing, observational, international study of the safety and effectiveness of ataluren in routine care. It is the first drug registry for patients with nmDMD that will provide real-world evidence regarding the long-term use of ataluren. Upon enrollment, patients will be followed up for a minimum of 5 years. The true effectiveness of ataluren was assessed by comparing results from the STRIDE registry (receiving both ataluren and standard of care (SoC)) and the Cooperative International Neuromuscular Research Group (CINRG) Duchenne Natural History Study (DNHS) (only receiving SoC) [118,119]. As of the cut-off date of 20 September 2021, the STRIDE patient registry demonstrated that treatment with ataluren delays loss of ambulation by more than five years in boys with nmDMD compared to SoC alone, and pulmonary function decline was also delayed by 1.8 years in those treated with ataluren and SoC [120]. Additionally, ataluren therapy was also beneficial in non-ambulatory nmDMD patients, which preserved lung function [117]. Recently, the EU label update supports ataluren use in patients who became non-ambulatory while on therapy [121]. The clinical benefit of ataluren in the treatment of nmDMD has also been demonstrated in several published case studies and retrospective case-series studies [122,123].

A 28-week clinical phase II study (NCT02758626) of ataluren (12-week treatment phase) in 7 subjects with DS and 8 subjects with CDD showed ataluren was not effective in reducing seizure frequency or improving cognitive, motor, or behavioral function or quality of life in subjects with either DS or CDD. The authors suggest that this may be due to (1) ataluren’s inability to cross the blood-brain barrier, (2) insufficient treatment duration for an effect, (3) selection of insensitive outcome measures, or (4) challenges related to protocol variances [124]. An in vitro experiment conducted simultaneously with this clinical trial showed that neither ataluren nor GJ072 was able to induce any detectable *CDKL5* gene readthrough in HEK293 cells transfected with the R59X and R134X CDKL5 constructs, therefore questioning the opportunity to use ataluren for the treatment ofnmCDD, but all tested *CDKL5* nonsense mutations are efficiently readthrough by G418 and gentamicin. The restored full-length CDKL5 proteins fully recovered their subcellular localization but only partially rescued their catalytic activity [125].

Furthermore, a phase II clinical trial (NCT02647359) evaluated the effect of ataluren in patients with nonsense mutation aniridia. It did not meet its primary endpoint of Maximum Reading Speed as measured by Minnesota Low Vision Reading Test (MNREAD) after 48 weeks of treatment, but the results did show a trend in favor of ataluren [126]. Due to the sponsor’s decision, the clinical trial of ataluren in hemophilia A and B was terminated early with no adverse safety findings. Clinical trials of ataluren in hemophilia and methylmalonic acidemia have been discontinued for low enrollment and unclear pharmacologic effects in available pharmacodynamic data (not due to any safety concerns).

Initially, ataluren was identified as a nonsense codon suppressor because it significantly increases FLuc activity in the PTC-containing FLuc reporter gene [85]. However, several studies found ataluren and related analogs are potent FLuc inhibitors that increase cellular FLuc activity levels by posttranslational stabilization instead of inducing PTC readthrough, that is, the activity of ataluren may be attributed to the off-target effect on the FLuc reporter used in the development of the molecule [127,128]. Ataluren could not induce any readthrough activity in a PTC-containing Renilla luciferase cell-based system and a diverse array of reporter assays [127,129]. In addition, ataluren has been reported to lack PTC readthrough activity in several disease models harboring nonsense mutation, such as frontotemporal dementia [130], long-QT syndrome type 1 [131], and monogenetic obesity [132]. Undeniably, ataluren does effectively promote PTC readthrough in certain conditions. Further studies are needed to elucidate the exact MOA of ataluren to fully understand the biased action of this compound for some PTC contexts and diseases.

### 3.4. Triterpenoid Saponin Compound-Escin

Escin is a natural mixture of triterpene saponins extracted from the horse chestnut tree with anti-edematous, anti-inflammatory, and venotonic properties, which has been clinically approved for treating various diseases, such as blunt trauma injuries and chronic venous insufficiency without known side effects [133]. Escin was identified as a readthrough agent for the CFTR-PTC mutations from 1600 clinically approved compounds in 2016 using two complementary HTS systems, including Fischer rat thyroid cells that were stably transfected with a dual-luciferase cDNA containing a UGAC or CFTR-G542X sequence (assessing molecular readthrough, Figure 2C) and transepithelial chloride conductance assay (evaluating CFTR functional rescue) [93]. Seven other hit compounds were also obtained using this screening strategy, including colchicine, cyclohexamide, pyranoradine tetraphosphate, prednicarbate, oxibendazole, doxorubicin and potassium paraamino benzoic acid, and escin showed the highest levels of readthrough, cell surface CFTR expression and forskolin-dependent short circuit current (Isc) alone and in combination with ivacaftor. It restored functional CFTR activity in a dose-dependent manner. Furthermore, escin enhanced CFTR mRNA expression levels approximately two-fold, suggesting that it also can inhibit NMD [93]. Future needs to verify the true therapeutic effect of escin in more diseases with a nonsense mutation. Developing PTC readthrough agents from clinically used drugs is an attractive strategy that allows us to evaluate promising compounds in humans rapidly.

### 3.5. Azoxanthone Drug-Amlexanox

Amlexanox is an azoxanthone medication having anti-allergic and anti-inflammatory effects that have been used therapeutically for over 30 years to treat canker sores, aphthous ulcers, and asthma [134]. Recently, it was found to have the potential to treat diabetes and obesity [135]. In 2012, a tethering-based screening system identified amlexanox as an NMD inhibitor from 1200 marketed drugs. Subsequently, the authors characterized amlexanox and found that it not only increased the amounts of PTC-containing mRNAs in *TP53*, *DMD*, and *CFTR* gene but also induced the synthesis of the corresponding functional full-length proteins, suggesting that amlexanox also could promote PTC readthrough [136]. Importantly, amlexanox treatment does not appear to affect the regulation of natural targets of NMD, such as Nat9, Tbl2, and SC35 [136]. A study showed that amlexanox inhibits NMD by reducing the phosphorylation of UPF1 [137], but another study showed the opposite result [138], and the specific MOA of amlexanox needs to be further explored. Based on the duality of amlexanox in inhibiting NMD and inducing PTC readthrough, the therapeutic potential of other genetic diseases with the nonsense mutation was further studied, including REDB [138], Aspartylglucosaminuria (AGU) [139], Primary ciliary dyskinesia [83], Bardet-Biedl syndrome and Alström syndrome [87], all of which show promising preliminary results. When compared to several other known PTC readthrough agents, the readthrough efficiency induced by the amlexanox appears to be better, such as 1 > Treatment with 25 μM amlexanox increased CFTR activity by almost three times more than treatment with 400 μM G418 or 25 μM ataluren [136]; 2 > readthrough of several *COL7A1* PTC induced by 250 μM amlexanox was more effective than 1 mM gentamicin [138]; 3 > 25 μM amlexanox significantly increased the enzyme activity in the AGU patient cells compared to 17 μM ataluren and 100 μg/mL G418 [139]. Interestingly, the combination of amlexanox and ataluren has a better therapeutic effect in CF, possibly because inhibition of NMD allowed more transcript availability for nonsense suppression [136]. Because amlexanox has been used clinically, together with these positive preclinical studies, suggests that amlexanox may be a highly promising therapy option for patients with nonsense mutations. Before it can be used in clinical settings, amlexanox must undergo extensive safety and efficacy research.

### 3.6. Amide Compounds-CC-90009, CC-885 and SJ6986

eRF3a participates in translation termination. Antisense oligonucleotides (ASOs) targeting eRF3a promoted modest PTC readthrough in hFIX-R338X hemophilia mice [140], suggesting the potential of targeting eRF3a for the treatment of genetic diseases caused by a nonsense mutation. Recently, two groups investigated the readthrough activity of several known eRF3a degraders, including small molecules CC-90009, CC-885, and SJ6986, which are amide compounds [141,142]. CC-90009 and CC-885 not only reduced eRF3a levels but also caused the reduction of eRF1 and increase of UPF1 levels, similar to the effects of eRF3 siRNA [142]. The study found CC-90009 and CC-885 promoted PTC readthrough by reducing translation termination and suppression of NMD. In HDQ-P1 cells and cells derived from four patients with different genetic diseases, CC-90009 or CC-885 alone did not induce or only induced a small amount of full-length protein synthesis, but the combination of low concentration G418 or gentamicin with CC-90009 or CC-885 strongly enhanced PTC readthrough. Based on this, the author proposed a ‘triple blow’ model-a strong PTC readthrough may be elicited when reduced levels of the translation termination factors, NMD inhibition, and conformational change of the ribosomal A site were achieved simultaneously. CC-90009 was less potent than CC-885 but was less cytotoxic and therefore showed a better activity window [142]. Notably, small-molecule eRF3a degraders have been found to have potent tumoricidal activity against acute myeloid leukemia (AML) cells [143]. CC-90009 displayed higher selectivity towards eRF3a with minimal to no effect on the rest of the proteome and is currently being used in clinical trials for AML treatment (NCT02848001) [144,145]. Another study showed that CC-90009 and SJ6986 rescued W1282X-CFTR function to approximately 20% and 5% of WT levels, respectively, and no synergistic effects of G418 and CC-90009 in rescuing the W1282X-CFTR were observed. CC-90009 or SJ6986 alone did little to rescue the G542X-CFTR function. However, when combined with G418, they both rescued G542X-CFTR function to approximately 50% of WT levels. The sensitivity and synergy between the eRF3a degraders and G418 appear to be highly dependent on mutation context [142]. Unexpectedly, CC-90009 or SJ6986 treatment inhibited ENaC expression and function. Off-target inhibition of ENaC may explain the adverse hypotension observed in CC-90009 clinical trials [142]. A potential concern is whether these eRF3 degraders would elicit readthrough at normal stop codons and further studies are required to address this issue.

### 3.7. Nucleoside Compounds

#### 3.7.1. Clitocine

Clitocine, a natural adenosine nucleoside analog first extracted from the mushroom *Clitocybe inversa* in the 1980s, is another PTC readthrough compound identified from the same HTS systems as ataluren [146]. In the nonsense luciferase reporter gene, the readthrough activity of clitocine was two to three orders of magnitude higher than that of gentamicin and G418. It showed the highest readthrough efficiency in UAA, followed by UGA, and then UAG. Unlike ataluren and aminoglycosides that act directly on the translation machinery, clitocine is incorporated into mRNA by RNA polymerase during transcription, substituting for adenosines. eRF1 is likely to weakly recognize PTCs with the incorporated clitocine, preventing effective termination and thereby increasing the chance of near-cognate tRNA recruitment [146]. Initial experiments had shown that clitocine does not disrupt sense codon decoding, indicating that, like adenosine, clitocine is preferentially base-paired with uracil to recruit cognate tRNA. Importantly, clitocine exhibited a marked preference for the readthrough of PTCs over that of normal stop codons. Clitocine induced the production of full-length p53 in a dose- and time-dependent manner in cell lines carrying nonsense mutations of *TP53*, and produced P53 was functional, which activated p53 target gene p21^waf1^ and drove cells into apoptosis. Furthermore, it inhibited the growth of CAOV-3_p53-UAA136_ xenograft tumors in mice [146]. Interestingly, several other studies suggested that clitocine can induce apoptosis in multidrug-resistant human cancer cells by inhibiting Mcl-1, which negatively regulates apoptosis by interacting with pro-apoptotic proteins such as Bak or Bax [147,148]. These data indicate that clitocine may be a molecular targeting strategy for cancer therapy. However, clitocine is unsuitable for development as a PTC readthrough agent for genetic diseases carrying nonsense mutations because of its high toxicity.

#### 3.7.2. H7 and 2,6-DAP

The HTS systems previously developed to identify readthrough-promoting molecules use cDNA constructs bearing nonsense mutations. The encoded mRNA does not undergo splicing, thus escaping NMD, which is inconsistent with what happens naturally in patients’ cells. Most human genes undergo splicing before being translated into proteins [149]. Spliced endogenous PTC-containing mRNAs in patient cells are subject to NMD, greatly reducing the amount of PTC-mRNA available for a readthrough. Therefore, using an un-spliced reporter gene is unlikely to accurately reflect the protein activity level restored by the readthrough of the endogenous transcripts. In 2017, Benhabiles et al. described a novel HTS system based on an NMD-prone mRNA for identifying molecules capable of effectively promoting PTC readthrough in human cells, which reproduced the fate of PTC-containing mRNAs in patient cells. It introduced a chimeric β-globin/immunoglobulin intron sequence into the cDNA sequence of firefly luciferase carrying PTC to make the corresponding mRNA subject to NMD (Figure 2D). Thus, only compounds that simultaneously inhibit NMD and activate readthrough, or very strongly activate readthrough of PTC-mRNAs having escaped NMD, can correct nonsense mutations on spliced mRNAs of this system [94].

Benhabiles et al. used the system to screen a library of 164 extracts of fungi and marine invertebrates. They found that mushroom *Lepista inversa* extract (named H7) corrected UGA and, to a lesser extent, UAA nonsense mutations but not (or very poorly) the UAG nonsense mutation. In the Calu-6 cell line (the *TP53* gene has a UGA nonsense mutation) and cells from CF patients, H7 promoted the synthesis of functional p53 and CFTR proteins, and H7 (25 ng/μL) has higher efficacy than G418 (1000 ng/μL). However, H7 was significantly less toxic than G418 at the concentrations used to induce readthrough. Interestingly, H7 does not inhibit NMD, suggesting that it can induce the synthesis of abundant full-length protein from the PTC-containing mRNAs that escape NMD [94]. Subsequently, they identified two molecules responsible for the readthrough activity of the H7 extract, one being the previously reported clitocine and the other 2,6-diaminopurine (DAP). DAP is also a naturally occurring adenine (A) analog and is reported to exert antiviral, antileukemia, and miRNA inhibition activity [150]. Commercial DAP was exclusively corrected for UGA nonsense mutations, and it is the first molecule found to correct only one stop codon [150]. In Calu-6 cell lines, DAP dose-dependently promoted the synthesis of functional full-length P53 proteins, and it has higher readthrough activity than G418. Furthermore, orally administered DAP strongly impaired Calu-6_p53-UGA_ xenograft tumor growth in mice as compared to DMSO. As with H7, DAP does not inhibit NMD and shows low toxicity [150]. Notably, the mechanism of DAP promoting readthrough is different from clitocine. FTSJ1 methyltransferase is an enzyme responsible for the posttranscriptional modification of certain tRNAs, including tRNA^Trp^ involved in UGA readthrough [151]. The study found that DAP reduced Cm34 methylation in the tRNA^Trp^ anticodon loop by inhibiting the activity of the FTSJ1 methyltransferase, thereby increasing the capacity of tRNA^Trp^ to recognize the UGA. After DAP treatment, only tryptophan was found to be incorporated into the UGA, making DAP especially suitable for correcting mutations that convert tryptophan-encoding codons to UGA [150]. The latest research found that DAP can correct various UGA mutations of the *CFTR* gene and restore its function in the CF mouse model, organoids derived from murine or patient cells, and CF patient cells [152]. In short, these results suggest that DAP is an excellent candidate drug for treating genetic diseases caused by UGA nonsense mutations. Future needs to verify the actual therapeutic effect of DAP in more diseases with nonsense mutation.

#### 3.7.3. SRI-37240 and SRI-41315

In 2021, Sharma et al. built another NMD-sensitive HST system consisting of the fusion of a PTC (UGAA)-containing NanoLuc cDNA at codon W134 with a human β-globin containing two intron sequences (Figure 2E). Using this system, they identified 180 compounds with potential readthrough activity from 771,345 compounds, of which SRI-37240, a nucleoside analog, was the most active compound. SRI-37240 restored the expression and function of full-length CFTR in vitro and synergized with G418. Moreover, it showed considerable selectivity for the readthrough of PTCs over normal stop codons [95]. SRI-37240 derivative SRI-41315 had better physiochemical features and readthrough activity. Compared to SRI-37240, SRI-41315 more efficiently restored CFTR function in CFTR-G542X HBEge cells, and G418 further enhanced its activity. Further mechanistic studies showed that SRI-41315 diminished eRF1 protein abundance through a proteasomal degradation-dependent pathway, which induced a prolonged translational pause at PTCs, leading to near-cognate amino acid insertion and thereby promoting readthrough. SRI-41315 is the first discovered drug that induces readthrough by affecting eRF1 levels [95]. Interestingly, ASO-mediated knockdown of eRF1 was also shown to augment translational readthrough in hFIX-R338X hemophilia mice [140]. These results suggest that eRF1 is another important target for PTC readthrough therapy. Unfortunately, SRI-37240 and SRI-41315 had undesirable effects on sodium transport by ENaC other than CFTR, which limits their potential to develop into CF therapeutic drugs [95], but they may be helpful in other disease models that are caused by PTCs.

#### 3.7.4. 2-Guanidino-Quinazoline

In 2022, Bidou et al. developed a three-step workflow strategy based on complementary assays to screen for effective read-through compounds. For the initial screening, they constructed an NIH 3T3 cell line stably transfected with a secreted Metridia luciferase cDNA interrupted by the *TP53* R213X mutation. This reporter gene is easy to measure, greatly simplifying the screening procedure, but is susceptible to false-positive hits because any molecule that increases the production (mRNA transcription, stability, translation) or secretion of Met-luciferase will be identified. In the secondary screen, they built a dual-reporter system based on different enzyme activities, which can eliminate many of the false-positive hits selected during the initial screen. Finally, they used a human HDQ-P1 cell line carrying an endogenous PTC mutation in the *TP53* gene to assess the effect of the drugs (Figure 2F) [96]. After three successive assays, they identified a new molecule, TLN468, from 17,680 initial molecules. TLN468 is a 2-Guanidino-quinazoline, which is named translectin. It showed a low level of toxicity between 20 and 60 μM and moderate toxicity for 80 μM. Significantly, TLN468 specifically stimulates PTC readthrough without altering the normal termination process. TLN468 promoted PTC readthrough in a dose-dependent manner. It increased *TP53* mRNA and restored functional full-length P53. Furthermore, TLN468 induced multiple DMD PTC readthrough with different context sequences. Interestingly, the readthrough activities of gentamicin and TLN468 had significant additive effects, suggesting that they might not bind to the same site [96]. 2-Guanidino-quinazolines has been described as bacterial translation inhibitor with antibacterial activity [153]. More work is needed to explain how TLN468 promotes PTC readthrough. Recently, another group has studied the SAR of TLN468. They synthesized a series of TLN468 derivatives and found that the key structure for their activity was a biguanide quinazoline/pyrimidine core. The compounds in the pyrimidine series surpassed those explored in the quinazoline series in terms of potency. A tertiary amine with aliphatic, hydrophobic substituents linked to the terminal guanidine nitrogen at approximately a 3-carbon distance led to significant potency increases. Further potency gains were obtained by flanking the pyrimidine ring with hydrophobic substituents, inducing readthrough at concentrations as low as 120 nM [154].

#### 3.7.5. 5-Fluorouridine (FUr)

Recently, Palomar-Siles et al. identified the clinically used anticancer drug 5-Fluorouracil (5-FU) as a potential *TP53* PTC readthrough-inducing agent by analyzing data available in the NCI-60 database [155]. 5-FU induces full-length p53 in R213X mutant *TP53* cells primarily through 5-Fluorouridine (FUr), whereas the other metabolite, 5-Fluoro-2′-deoxyuridine (FdUr), has little readthrough activity. FdUr is thought to exert cytotoxic effects by incorporating it into DNA and inhibiting thymidylate synthase [156]. Full-length p53 produced by FUr was transcriptionally active and can trigger cell death by apoptosis. Furthermore, FUr also increased full-length p53 levels in xenograft tumors with R213X *TP53* nonsense mutation. Compared with G418, a greater proportion of FUr-induced readthrough occurred at PTCs than at normal stop codons [155]. Interestingly, FUr and FdUr also induced wild-type p53, consistent with the previously reported that 5-FU can stabilize WT p53 [157], indicating FUr may increase the level of full-length p53 protein by both inducing translational readthrough and stabilizing p53 protein simultaneously. One possible mechanism for FUr-induced translational readthrough is that FUr replaces uridine in mRNA, similar to clitocine. FUr incorporated into mRNA can potentially base-pair with guanine, allowing insertion of Arg tRNA at the *TP53* R213X UGA, thereby producing full-length P53. Based on the ability of FUr to restore functional P53 protein, tumor patients with nonsense mutations of *TP53* may be more sensitive to chemotherapy with 5-FU. In addition, it is interesting to explore FUr itself as a therapeutic agent for tumor patients with nonsense mutation *TP53*, which may reduce the side effects associated with FdUr-induced DNA damage. In addition to *TP53*, FUr could also promote readthrough of other cancer-related genes PTC, such as *APC*, *PTEN*, *RB1*, and *TET2*, which require further study [155].

### 3.8. Other Non-Aminoglycoside TRIDs

In 2009, Du et al. developed a sensitive and quantitative luciferase-independent HTS system, protein transcription/translation (PTT)–ELISA, which was driven by a plasmid containing ATM gene fragment (coding sequence for 403-1986aa) with a TGA C mutation to identify novel PTC-readthrough compounds (Figure 2G) [97]. Initially, they used the system to screen ~34,000 compounds and identified 12 low-molecular-weight compounds with potential readthrough activity, of which RTC13 and RTC14 showed the ability to induce PTC readthrough in A-T cell models and *mdx* mouse myotube cells with similar readthrough efficiency to G418 and gentamicin [97]. Their toxicity in mammalian cells was lower, and do not significantly affect normal stop codons [97]. In *mdx* mice, intramuscular injections of RTC13 promoted readthrough of the *mdx* UAA stop codon more efficiently than gentamicin, ataluren, or RTC14. When administered systemically, RTC13 partially restored dystrophin function in all major muscle groups, including the diaphragm and heart, improving muscle function and decreasing creatine kinase (CK) levels [158]. RTC13 is well tolerated in mice and does not produce toxicity after repeated administration at 30 mg/kg [158]. Furthermore, the readthrough effect of RTC13 was also confirmed in more than 20 pathogenic nonsense mutations of the collagen VII gene [159]. RTC14 increased mRNA expression of the *XPC* gene and *NAGLU* gene in fibroblasts from xeroderma pigmentosum group C and Sanfilippo C patients, respectively [160]. However, RTC13 or RTC14 could not suppress PTCs associated with hemophilia A, McArdle disease, chronic granulomatous disease, and Wiscott-Aldrich syndrome [159,161,162,163]. A study of the SAR of RTC13 revealed that several derivatives containing variations on both aryl and thiazolidinone rings exhibited good readthrough activity. Moreover, the RTC13 derivative of thiazolidinone rings substituted by pyrimidinedione units also showed excellent readthrough activity [164].

In 2013, Du et al. identified four novel PTC readthrough compounds, GJ071, GJ072, RTC204, and RTC219, from an additional ~36,000 small molecular weight compounds using the same HTS system. GJ071 and GJ072 can induce readthrough of all three PTCs of the ATM gene in A-T patient-derived lymphoblastoid cell lines [165]. Subsequently, the SAR of GJ071 and GJ072 was further studied, and several GJ072 analogs showed similar readthrough ability to GJ072. One of these analogs, GJ103, has developed a water-soluble salt form, which allows systematic administration to evaluate it is in vivo activity [165,166]. RTC204 and RTC219 shared similar structural features to GJ072 and its analogs, suggesting that this feature may be responsible for their readthrough activity [165]. In the A-T cell models, GJ072 and its analog GJ103 showed a similar readthrough activity to RTC13 (both superior to ataluren) but were more tolerable than RTC13 and RTC14 [165]. The mechanisms of action of RTC13 and GJ072 are likely similar to that of ataluren [24].

Recently, Tutone et al. identified four TRIDs with potential readthrough activity through a pharmacophore-based modeling study, including NV2899, NV2909, NV2913, and NV2907. These molecules do not contain the oxadiazole core, show a low cytotoxic effect, and are considered potential lead compounds for TRIDs [167].

## 4. Conclusions and Perspectives

TRIDs have always been the research focus of PTC readthrough therapy due to their potential therapeutic advantages. To date, more than 50 TRIDs have been found, which can be divided into two major categories: aminoglycosides and non-aminoglycosides. Gentamicin is the only aminoglycoside entering clinical trials, which has potential therapeutic benefits for CF and genodermatosis [29,36]. However, it may not be suitable for long-term use as a readthrough drug due to potential ototoxicity and nephrotoxicity. Several methods for reducing the toxicity of aminoglycosides by co-administration with other molecules have been proposed, and further research is needed [6]. In addition, through reasonable modification/design of aminoglycoside structure, several aminoglycoside derivatives with reduced toxicity and retained or increased readthrough activity have been developed. ELX-02, the most representative aminoglycoside derivative, is considered to be a promising non-toxic alternative for G418 and has entered clinical trials for CF, nephropathic cystinosis, and Alport syndrome [62]. Ataluren is the most studied non-aminoglycoside capable of inducing PTC readthrough at present. It has been conditionally approved by the EU and some other countries to treat nmDMD and is the only commercially available TRID [116]. However, other results see this compound as controversial and further studies are needed to elucidate the exact MOA of ataluren [4]. Over the last few years, approximately 15 novel non-aminoglycoside TRIDs have been identified through various HTS systems. Some compounds, such as amlexanox, escin, 2,6-DAP, and TLN468, showed good PTC readthrough efficiency. The most commonly used TRIDs in the proof-of-concept studies for PTC readthrough therapy are G418, gentamicin, and ataluren. So far, about 100 studies (Supplementary Table S1) have shown that TRIDs have potential therapeutic benefits for more than 40 genetic disorders caused by nonsense mutation, including cancer. However, long-term safety, effectiveness, precise MOA, the exact identity of the amino acid inserted at the PTC position, pharmacodynamics, and pharmacokinetics studies are required before the clinical use of these compounds becomes possible.

The critical factor determining the effectiveness of PTC readthrough therapy is restoring the minimal amount of the functional full-length protein required for phenotype alleviation. For most inherited diseases, the therapeutic threshold is largely unknown [14]. Current TRIDs can only partially restore the full-length protein’s expression, which is inadequate for treating genetic diseases with a high threshold for correction. Therefore, it is necessary to find novel TRIDs with high effectiveness and lower toxicity, either through modifying existing TRIDs or discovering completely new compounds with enhanced PTC readthrough efficiency. The combined use of multiple TRIDs may increase PTC read-through efficiency, and further research is needed to determine optimal TRIDs combinations. Drug-induced PTC readthrough efficiency is not only determined by TRIDs themselves but also influenced by PTC identity, PTC context sequence, NMD efficiency, TRIDs bioavailability, and other factors [6]. It is important to note that not all nonsense mutations can be induced to undergo readthrough. Therefore, a thorough assessment of readthrough susceptibility of disease-related PTC in each patient is necessary for personalized treatment. In addition, the specific amino acid incorporated during PTC decoding plays an essential issue in PTC readthrough therapy. Previous studies have shown that Arg, Trp, and Cys are most likely to insert the UGA codons while Gln, Lys, and Tyr are the predominant choices for UAA and UAG codons [98,168]. Amino acids different from normal proteins are likely introduced into PTC during readthrough, i.e., missense mutation occurs. If the gene is intolerant to missense mutation, its normal functional activity will be damaged even if the full-length protein is produced after PTC readthrough. Further research is needed to gain a deep understanding of the mechanism of eukaryotic termination, recoding, and drug-induced PTC readthrough to maximize the benefits of various TRID treatments for patients.

## Figures and Tables

**Table 1:** Efficacy of Transdermal Gentamicin Administration in Treating Hereditary Skin Diseases.

Disease	Gene	Administration	Dose and Duration	Results
EBS-MD [26]	*PLEC1*	Intravenous	*n* = 1; 7.5 mg/kg/d for 14 consecutive days; 2 treatment courses	Increased expression of plectin in the skin for at least 5 months, myalgia disappeared, and quality of life improved
JEB [25]	*LAMA3*/ *LAMB3*	Intravenous	1. *n* = 3 (7.5 mg/kg daily for 14 days); 2. *n* = 2 (10 mg/kg daily for 24 days)	All 5 patients exhibited increased laminin 332 in the dermal-epidermal junction, and improved wound closure
JEB [45]	*LAMB3*	Intravenous	*n* = 5; 7.5 mg/kg/d for three weeks	a positive impact on skin fragility and daily life in four patients
JEB [42]	*LAMB3*	Topical	*n* = 1; 0.3% gentamicin ointment once at bedtime	Conjunctival cells showed positive staining for laminin-332, and the amelioration of the corneal erosions,
NPPK [38]	*SERPINB7*	Topical	*n* = 20; 0.1% or 0.3% gentamicin ointment once daily for 30 days	Significantly improve hyperkeratosis and foul smell
GS-JEB [40]	*LAMA3*/ *LAMB3*	Topical	*n* = 3; 0.5% gentamicin ointment twice a day for 2 weeks	Increased expression of laminin 332 at the dermal-epidermal junction for at least 3 months, and improved wound closure.
JEB-gen intermed [41]	*COL17A1*	Topical	*n* = 1; 0.3% gentamicin ointment once daily for 90 days	Improved wound closure and reduced blister formation. After drug withdrawal, there was no relapse for at least 2 months.
HSS [44]	*CDSN*	Topical	*n* = 4; 0.1% gentamicin ointment twice a day for 6 months	Significant hair growth and SALT score reduction
NPPK [37]	*SERPINB7*	Topical	*n* = 5; 0.1% gentamicin ointment twice a day for 4 weeks	Suppressed hyperkeratosis did not improve the degree of erythema
RDEB [39]	*COL17A1*	Topical; intradermal injection	*n* = 5; 0.1% gentamicin ointment 3 times daily for 2 weeks; 8 mg for 2 days	Both induce type VII collagen and anchoring fibrils; Topical: corrected dermal-epidermal separation, improved wound closure, and reduced blister formation.
HHD [43]	*ATP2C1*	Topical	*n* = 1; 0.1% gentamicin ointment twice a day for 18 days	Far more effective in inducing remission in an HHD patient than an accepted topical disinfectant

EBS-MD: Epidermolysis bullosa simplex with muscular dystrophy; JEB: Junctional epidermolysis bullosa; NPPK: Nagashima-type palmoplantar keratosis; GS-JEB: Generalized severe junctional epidermolysis bullosa; HSS: hereditary hypotrichosis simplex of the scalp; RDEB: Recessive dystrophic epidermolysis bullosa; HHD: Hailey-Hailey disease.

**Table 2:** Summary of preclinical studies on Ataluren as a PTC suppressor in patient-derived fibroblasts, iPSCs, and organoids models over the past 10 years.

Disease	Gene	Model	Conclusion
Leber congenital amaurosis type 4 [103]	*AIPL1*	Retinal organoids	The increased level of full-length AIPL1 protein mediated by ataluren readthrough was not sufficient to restore rod PDE6 to the levels required to reduce cGMP
Retinitis pigmentosa [104]	*FAM161A*	Fibroblasts from six patients	Ataluren was able to restore FAM161A expression in FAM161A-mutated cells as well as its co-localization with α-tubulin along the microtubules.
Bardet-Biedl syndrome; Alström syndrome [87]	*BBS2*; *ALMS1*	Patient-derived fibroblasts	Ataluren treatment recovered full-length BBS2 or ALMS1 protein expression and ciliary function.
Retinitis pigmentosa [105]	*RPGR*	Patient-derived fibroblasts	Applying ataluren restored RPGR at the cilium in approximately 8% of patient-derived cells
Usher syndrome [106]	*USH2A*	Patient-derived fibroblasts	Ataluren increased USH2A protein expression and the number of ciliated cells
Inherited retinal dystrophies [107]	*CHM*	Patient-derived fibroblasts; Patient iPSCs -derived RPE	Ataluren treatment induced a non-significant trend for functional rescue, which could not be improved by nonsense-mediated decay inhibition.
Lamin A/C (LMNA)-related cardiomyopathy [108]	*LMNA*	Patient iPSCs-derived cardiomyocytes	Ataluren treatment increased the production of full-length LMNA proteins in only the R225X mutant, not in other mutations.
Retinitis pigmentosa [109]	*MERTK*	Patient iPSCs -derived RPE	Following treatment with ataluren was able to restore the expression of MERTK. Furthermore, the ataluren treatment restored 12% of the phagocytic function.
Cystic fibrosis [110]	*CFTR*	Intestinal organoids	Functional restoration of CFTR by PTC124 could not be confirmed
Heart disease [111]	*SCN5A*	Patient iPSCs-derived cardiomyocytes	The authors did not observe the rescue of the electrophysiological phenotype in hiPSC-derived cardiomyocytes from the patients
Choroideremia [91]	*CHM*	Patient-derived fibroblasts	After treatment with ataluren, prenylation activity recovered, but no increase of REP1 protein was detected.
X-linked retinitis Pigmentosa [112]	*RP2*	Patient iPSCs -derived RPE	After treatment with ataluren, up to 20% of endogenous, full-length RP2 protein can be restored.
Neuronal ceroid lipofuscinosis [113]	*TPP1*	Patient iPSCs-derived neural progenitor cells	Nonsense suppression by PTC124 resulted in both an increase in TPP1 activity and an attenuation of neuropathology

RPE: retinal pigment epithelium.

**Table 3:** Summary of key clinical trials of Ataluren in nmDMD, including completed Phase II and III clinical trials and ongoing clinical trials.

Phase and Status	Duration	Age	Group and Dose	Results
IIa (NCT00264888) Completed	2005.12–2007.05	5 years and older	1. *n* = 6 (4, 4, 8 mg/kg/day) 2. *n* = 20 (10, 10, 20 mg/kg/day) 3. *n* = 12 (20, 20, 40 mg/kg/day)	61% of subjects demonstrated positive changes in dystrophin expression after 28 days of treatment; Changes in Clinical Measures were not statistically significant; Ataluren was generally well tolerated.
IIb (NCT00592553) Completed	2008.02–2009.12	5 years and older	1. *n* = 57 (10, 10, 20 mg/kg/day) 2. *n* = 60 (20, 20, 40 mg/kg/day) 3. *n* = 57 (placebo)	40 mg/kg/day slowed the rate of decline of walking ability (6MWD) after 48 weeks of treatment (ataluren performed better in a post-hoc analysis); 80 mg/kg/day had no activity; well tolerated.
III (NCT01826487) Completed	2013.03–2014.08	7–16 years	1. *n* = 115 (10, 10, 20 mg/kg/day) 2. *n* = 115 (placebo)	After 48 weeks of treatment, the change in 6MWD between ataluren-treated and placebo-treated patients in the intention-to-treat population was insignificant; but the benefit observed in patients with a baseline 6MWD of 300 m or more to less than 400 m supports the clinical benefit of ataluren.
III (NCT01557400) Completed	2012.05–2018.01	12.8 ± 2.4 years	Single Group Assignment *n* = 94 (10, 10, 20 mg/kg/day)	After 240 weeks of treatment, ataluren plus standard of care delays disease progression and benefits ambulatory and non-ambulatory patients with nmDMD
II (NCT02819557) Completed	2016.09–2018.09	2–5 years	Single Group Assignment *n* = 14 (10, 10, 20 mg/kg/day)	The safety and pharmacokinetic profile of ataluren in children from 2–5 years with nmDMD was consistent with that for older children; Clinical benefits were also observed at 52 weeks with ataluren.
III (NCT03179631) Ongoing	2017.07–2023.07	5 years and older	Randomized, double-blind, placebo-controlled	The primary outcome to be assessed is the change slope in 6MWD over 72 weeks. Its estimated completion date is July 2023.
NCT02369731 Ongoing	2015.04–2025.05	2 years and older	A long-term, multicenter, observational study	It evaluates the safety and effectiveness of ataluren in usual care. Its estimated completion date is May 2025.
II (NCT04336826) Ongoing	2021.12–2022.12	6 months to 2 years	An open-label study	It evaluates the safety and pharmacokinetics of ataluren over 24 weeks. Its estimated completion date is December 2022.

6MWD: 6-Minute Walk Distance.

## Data Availability

Not applicable.

## Supplementary Materials

The following supporting information can be downloaded at: https://www.mdpi.com/article/10.3390/biom13060988/s1, Supplementary Table S1: Summary of PTC readthrough activity of TRIDs (mainly ataluren G418 and gentamicin) in different non-clinical models of genetic disorders caused by nonsense mutations.
